# Measuring Medical Student Scholarly Activity After a Structured Plastic Surgery Research Fellowship: A Bibliometric Analysis

**DOI:** 10.1093/asjof/ojaf012

**Published:** 2025-02-21

**Authors:** Jasmine A Panton, Bhavana Thota, Abby J Culver, Jeffrey M Kenkel

## Abstract

**Background:**

Plastic surgery is consistently one of the most competitive medical specialties in the National Resident Matching Program match, with students often partaking in dedicated research fellowships to bolster their applications.

**Objectives:**

The purpose of this study was to quantify the academic productivity of medical students completing a structured plastic surgery research fellowship at a single academic center over a 7-year period.

**Methods:**

The bibliometric output of 26 medical student research fellows was analyzed. Eligible scholarly activities for analysis included peer-reviewed articles, books and book chapters, and video editorials published in the field of plastic surgery and indexed between July 1 of the fellowship and September 1, 2023. Cross-verified citation lists were generated for each author and *h*-index, publication number, number of citing articles, sum of times cited, author position, and journal or book title were recorded.

**Results:**

Twenty-four of 26 research fellows have published a total of 177 scholarly activities (93.22% articles) which have been cited in ∼322 articles. The mean publication per fellow is 6.69 and the mean *h-*index is 1.46. Analysis excluding the 2022-2023 cohort revealed a strong positive correlation between years since beginning research fellowship and both mean *h*-index (*r* = 0.84, *P* < .001) and mean number of publications per year (*r* = 0.81, *P* < .001).

**Conclusions:**

Structured research fellowships can serve as a springboard for medical students to increase research output and engage meaningfully with academia. The authors of this study suggest that publications in peer-reviewed journals continue to increase following participation in a structured plastic surgery research fellowship.

**Level of Evidence: 4 (Therapeutic):**

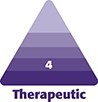

Medical students interested in applying to integrated plastic surgery residencies engage in research before the National Resident Matching Program (NRMP) match to strengthen their application and enhance their exposure to the field. Some choose to complete a dedicated research year before applying to increase the competitiveness of their application.^1,2^ Dedicated time for research in plastic surgery may allow for more meaningful scholarly outputs. Structured research fellowships allow participants to develop more meaningful relationships with mentors and enhance their knowledge about the breadth of plastic surgery before applying for residency.

Bibliometric data, such as the number of publications and Hirsch index (*h*-index),^3,4^ provide objective measurement of academic productivity^5^ and have been used to measure scholarly output among applicants,^1,6-8^ trainees,^9-12^ and faculty^13^ in plastic surgery.

To the authors’ knowledge, there is a paucity of data existing using bibliometric data to quantify the research output of medical students engaged in a formal research fellowship and track their subsequent scholarly impact in the years following. The purpose of this study was to quantify the academic productivity of medical students completing a dedicated, structured plastic surgery research fellowship at a single academic center. Additionally, we sought to measure continued productivity, scholarly output, and academic impact following completion of their fellowship.

## METHODS

### Design and Implementation of Structured Research Fellowship for Medical Students

Structured research fellowships differ from “unstructured” research fellowships in that medical students are paired directly with surgeon investigators through an application and interview process. Alternatively, many students find research opportunities by directly reaching out to potential mentors through email or social media.

At the study institution, students formally apply online, submitting basic demographic information, United States Medical Licensing Examination test scores, and supplemental essays to the department. Predilections for different subspecialties are indicated through a supplemental essay. Potential fellows are then interviewed by attendings focusing on research in various plastic surgery subspecialties ranging from aesthetics, hand surgery, craniofacial surgery, breast reconstruction, and microsurgery. A position is offered to the student several months in advance, attempting to match the student's interest with a faculty member's need.

**Figure 1.**
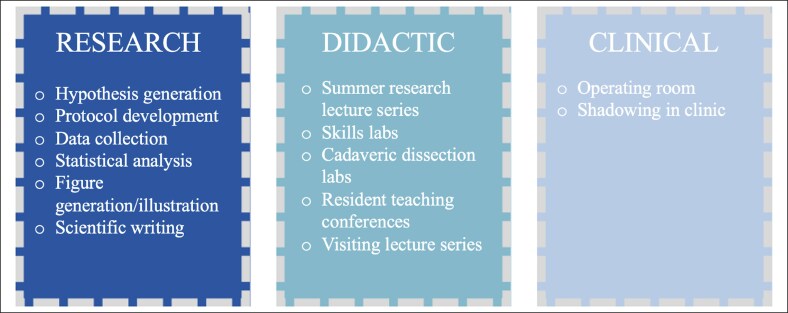
Clinical research fellowship structure.

**Figure 2.**
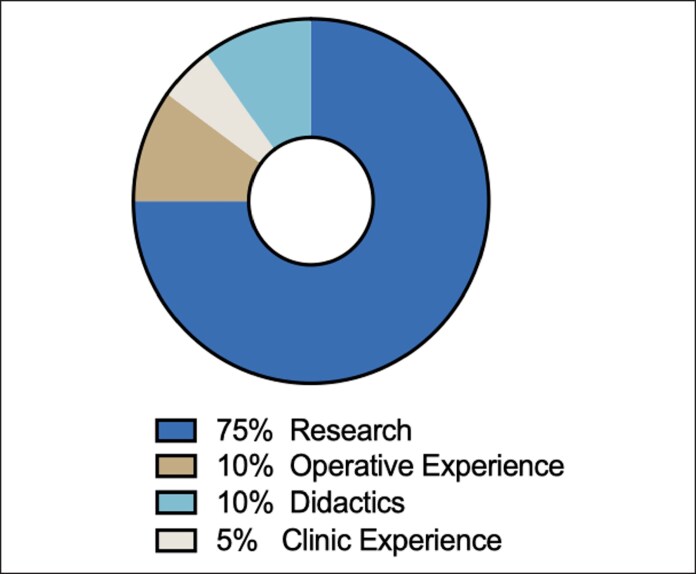
Time breakdown of clinical research fellowship.

Although the schedule varies among students, most begin their research fellowship the summer following their third year of medical school. During that summer, fellows attend a Summer Research Lecture Series hosted by faculty and research staff in the department, with lecture topics ranging from the aesthetic principles of aging to Gillies’ principles of plastic surgery. Adjunct skills laborateries provide dedicated time for fellows to practice surgical suturing and knot-tying, plan Z-plasty's, and dissect cadaveric hands. Throughout the year, fellows attend resident teaching conferences, including photographic conference, anatomy laboratories, and visiting lecture series, providing early exposure to the Accreditation Council for Graduate Medical Education curriculum they will experience as residents. Operating room time and clinical shadowing with both their own mentors and other faculty are encouraged to facilitate exposure to the breadth of the specialty (Figure 1). Over the course of the year, the breakdown is roughly 75% clinical or translational research, 15% didactic, and 10% clinical, but may vary according to the nature of the fellow's research (Figure 2).

The broad range of exposure—didactic, clinical, and operative—allows students to better understand the specialty and formulate appropriate investigative questions to be studied. The research fellows often continue work on the previous year's studies but also start to work on developing their own projects. Fellows work closely with dedicated departmental research coordinators to formalize research questions through protocols submitted to the IRB. Fellows meet consistently with mentors and faculty to define endpoints, study populations, and appropriate assessments. During IRB submission, fellows reinforce the importance of adhering to institutional and global ethical standards for evidence-based research.^14^

### Academic Productivity

A cross-sectional bibliometric analysis of publication, citation, and authorship records using Web of Science (Clarivate, Philadelphia, PA) (WoS) was performed in August 2024.^14^ Inclusion and exclusion criteria for analysis are outlined in Table 1. The academic productivity of 26 research fellows from a single academic center was analyzed. Public profiles on Open Research and Contributor ID (ORCID iD)^15^ ResearchGate,^16^ and Google Scholar^17^ were used to cross-reference peer-reviewed publications with authorship. After cross-verifying citation, lists were generated for each author, the following data were measured using the Citation Report feature of the Web of Science online portal: (1) total number of publications in plastic surgery; (2) number of citing articles; (3) total number of times cited; (4) total number of times cited without self-cite; (5) publication date; (6) journal or book title; (7) author position; and (8) *h*-index.

**Table 1.** Inclusion and Exclusion Criteria

Inclusion criteria	Exclusion criteria
1. Research fellows from 2016 to 2023 2. Peer-reviewed articles 3. Books and book chapters 4. Video editorials 5. Publications in open-access journals 6. Publications indexed between July 1 of the fellowship year and September 1, 2023 7. Publications authored by clinician investigators within the fellowship institution	1. Conference presentations, posters, and abstracts 2. Eligible publications published outside of the scope of plastic and reconstructive surgery 3. Publications published before the beginning research fellowship 4. Publications authored by clinician investigators outside the fellowship institution

The Hirsch, or *h-*index,^4^ was determined to measure the academic output objectively and its impact. Briefly, the authors have index *h* if *h* of their total *N_p_* papers have at least *h* citations each; additionally, the authors remaining publications (*N_p_*−*h*) must each have less than or equal to *h* citations.^3,4^

Peer-reviewed articles, books, book chapters, and video publications were included in this analysis. Articles published before beginning the research fellowship were excluded. Articles published following the fellowship duration on topics distinct from plastic and reconstructive surgery (eg, neurosurgery, nutrition) were excluded from analysis. Published conference abstracts, posters, and presentations were excluded from analysis because of the lack of citation data and variations in publishing practices between different journals. Duplicates among individual fellows were counted once; however, duplicate authorship between fellows (eg, 2 fellows contributing to 1 publication) was counted separately. To eliminate potential bias, fellows were not queried directly for publication records.^18,19^ Additionally, following sensitivity analysis, publications co-authored with faculty outside the institution in this study were considered to potentially be confounding and excluded from analysis. Other data included in the analysis were baseline characteristics of the research fellows, training level and specialty, and residency match rate, which were obtained through public profiles.

### Statistical Analysis

To quantify baseline characteristics of fellows and citation metrics, descriptive statistics were used. To trend the temporal relationship between years since the beginning research fellowship and academic output, a Pearson correlation analysis was performed. Additional analyses were performed between the cohort of fellows before and those after Summer 2020 to illustrate the relationship between bibliometric data and time. A *P-*value ≤.05 was considered statistically significant. Findings were reported according to the STROBE checklist for cross-sectional studies.^20^

## RESULTS

### Research Fellows

**Figure 3.**
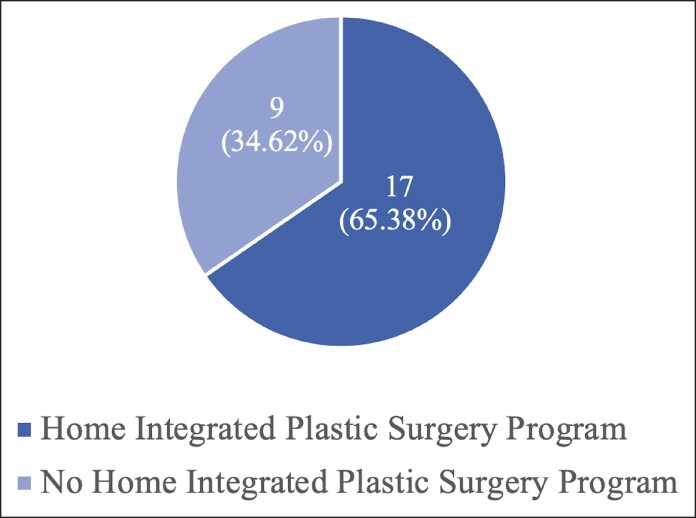
The presence of integrated plastic surgery residency program at research fellow medical institutions.

**Figure 4.**
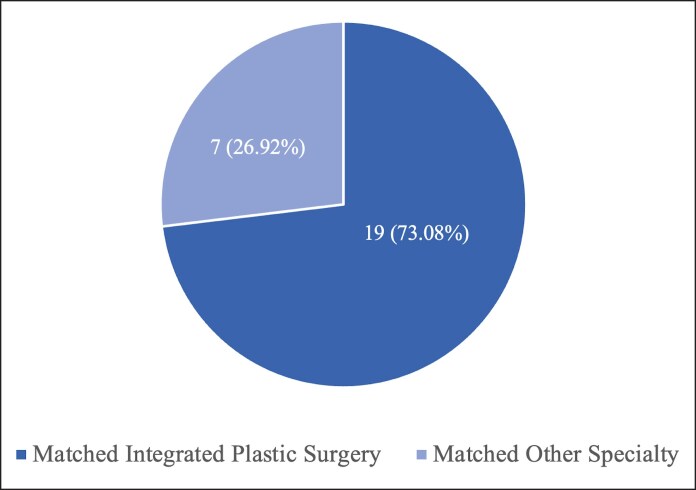
Research fellow match outcomes.

Descriptive statistics of research fellows are displayed in Table 2. Twenty-six fellows have participated in the past 7 years of the fellowship, all completing ≥1 year of research with their assigned mentor. Among them, 57.69% of the fellows were females, and 65.38% of fellows were from institutions with integrated plastic surgery residency programs (Figure 3). One fellow completed 2 years of research and was included in the cohort of fellows who began their investigative time before the summer of 2020. Fellows worked across plastic surgery subspecialties including aesthetics (*n* = 6); breast reconstruction and microsurgery (*n* = 5); pediatric and craniofacial (*n* = 7); facial reconstruction and microsurgery (*n* = 3); and hand (*n* = 5). All research fellows (*n* = 26, 100%) ultimately matched into a residency program, with 73.08% (*n* = 19) matching into integrated plastic surgery programs and the remaining 26.92% (*n* = 7) into other specialties (Figure 4).

**Table 2.** Research Fellow Characteristics

Research fellow characteristics	*n*	%
Research fellows	26	
Gender		
Female	15	57.69
Male	11	42.31
Area of research		
Aesthetic	6	23.08
Breast reconstruction and microsurgery	5	19.23
Craniofacial/pediatrics	7	26.92
Facial reconstruction and microsurgery	3	11.54
Hand	5	19.23
Length of research fellowship		
1 year	25	96.15
>1 year	1	3.85
Year of Fellowship		
2016-2017	2	7.69
2017-2018	2	7.69
2018-2019	1	3.85
2019-2020	6	23.08
2020-2021	4	15.38
2021-2022	6	23.08
2022-2023	5	19.23

### Publication and Citation Analysis

The publications and bibliometric data of the 26 research fellows were identified using WoS databases and citation reporting. Two craniofacial fellows did not have bibliometric data that met inclusion criteria. Their data were still incorporated into stratification, trends, and analysis.

**Figure 5.**
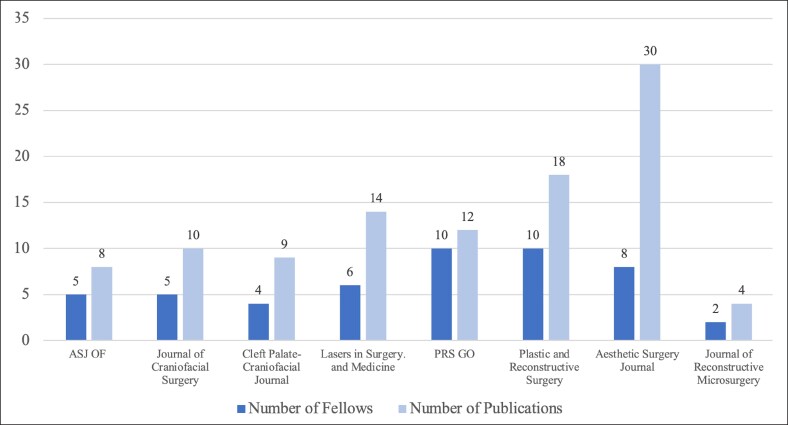
Research fellows’ contribution to scholarly journals in plastic and reconstructive surgery.

Descriptive publication and citation statistics are summarized in Table 3. In the last 7 years, 24 of 26 fellows have produced ≥1 publication, resulting in 177 publications (93.22% articles, *n* = 165) cited by ∼322 articles. In ≥1 publication, 76.92% of fellows contributed as first author. Mean publications per fellow was 6.69 (median 5.5, range, 0-35) for the cohort. Mean publications for fellows beginning before Summer 2020 was 11.0 and 3.73 for fellows beginning during or after Summer 2020. The maximum number of citations for a single fellow was 122 (min: 0) over a career and 36 (min: 0) over 1 year. The maximum number of citations for a single publication (min: 0) was 41. Mean *h*-index was 2.27 for fellows before Summer 2020 and 0.87 for fellows after. Fellows were published most frequently in the *Aesthetic Surgery Journal*, with 30 articles total. Published works additionally appeared in 12 other peer-reviewed plastic surgery journals. Fellows’ top contributions to individual journals are summarized in Figure 5.

**Table 3.** Publication and Citation Data

Publication data	*n*	%	Mean	Citation data	*n*	Mean
Publications	177		6.81	*h-*index		1.46
Articles	165	93.22		Citing articles	322	12.38
Book chapters	10	5.65		Times cited	339	13.04
Books	1	0.56		Times cited without self-cite	309	11.88
Editorial videos	2	1.13				
2016-2017	39	22.03	19.5			
2017-2018	14	7.91	7			
2018-2019	10	5.65	10			
2019-2020	58	32.77	9.67			
2020-2021	12	6.78	3			
2021-2022	23	12.99	3.8			
2022-2023	21	11.86	4.2			

### Trending Productivity

**Figure 6.**
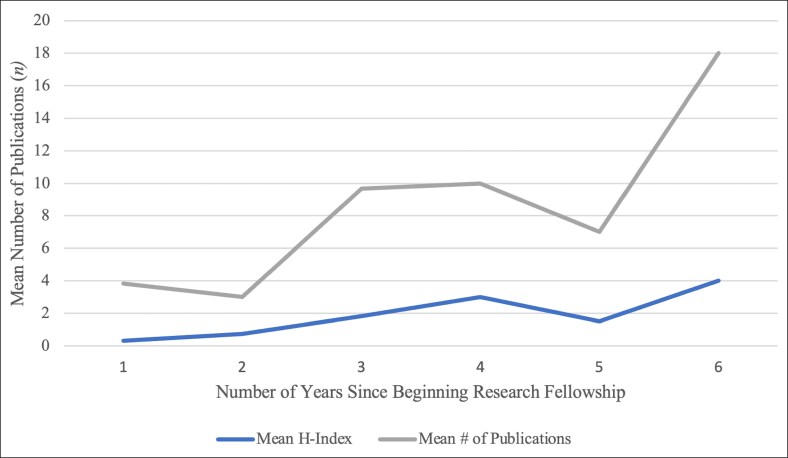
Scholarly output since research fellowship.

**Figure 7.**
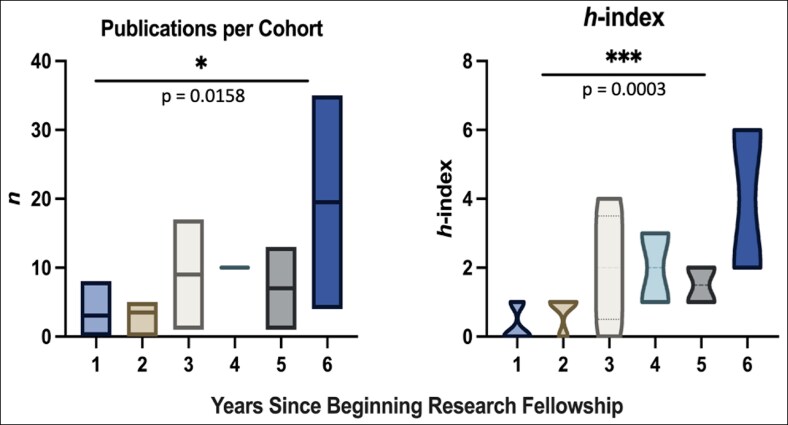
Total publications and *h*-index over years since beginning research fellowship.

The publication and citation analyses illustrated that the number of publications and citations increased as fellows advanced further in their careers (Figure 6). A strong positive correlation was identified between years since beginning research fellowship and both mean *h*-index (*r* = 0.84, *P* < .001) and mean number of publications (*r* = 0.81, *P* < .001; Figure 7). Additionally, there was a positive correlation between years since beginning research fellowship and total number of publications (*r* = 0.1, *P* = .65). This analysis excluded the most recent cohort of research fellows described within this study (2022-2023), because this group of research fellows just completed their fellowships in the summer of 2023 and have not progressed far enough into their careers to warrant such an analysis.

## DISCUSSION

Although there have been several studies examining the research productivity of integrated plastic surgery matriculants, there are few studies describing the research output and impact of medical students participating in a structured plastic surgery research fellowship.^6,8^ This study aims to analyze the value of a structured plastic surgery research fellowship at a single academic institution by measuring the research output of the fellows following completion of their dedicated research time.

According to a 2016 survey of the American Council of Academic Plastic Surgeons (ACAPS), program directors define a “productive” research year as 3 or 4 publications and presentations per fellowship year.^1^ The mean number of publications for the most recent cohort that completed their fellowship in the summer of 2023 was 4.2, slightly above the range of publications ACAPS considers to be competitive with a dedicated research time of 1 year.

Several research fellows had their work published in top-tier subspecialty journals such as the *Aesthetic Surgery Journal*. This highlights a unique aspect of the study institution's structured plastic surgery research fellowship in that students largely conduct research within a certain subspecialty within plastic surgery. Many structured plastic surgery research fellowships at other high-volume academic institutions do not allow for this degree of specialized research focus, with 1 prominent exception being craniofacial research fellowships.^21,22^ Certainly, the degree of emphasis on aesthetics research found within the study institution's structured plastic surgery research fellowship is unique, with 23.08% of the medical student fellows being dedicated to aesthetics research and the cohort producing a total of 30 publications in *Aesthetic Surgery Journal*, 14 publications in *Lasers in Surgery and Medicine*, and 8 publications in *Aesthetic Surgery Journal Open Forum*.^22^

The increase in mean publications over time, even after completion of the research fellowship, was largely expected. This could be because these students continue to produce scholarly work as surgical trainees, demonstrating an interest in research beyond that perceived to be necessary for building a residency application. It could also be attributed to completing projects begun during their dedicated time and/or delayed publication or revision cycles. Furthermore, trends in research productivity among the study institution's cohort of fellows revealed a strong positive correlation between years since beginning the research fellowship and both mean *h*-index (*P* < .001) and mean number of publications (*P* < .001). This finding highlights the impact of the research conducted by the fellows, in that their work has likely been cited more in the years since publication. This indicates that the fellows are making meaningful contributions to the plastic surgery literature, in addition to amassing a considerable number of publications.

The observed increase in the mean *h-*index as students’ transition from undergraduate to graduate medical education underscores the academic value of the research year; it corresponds to both an increase in citations and cited publications over time. This upward trend in impact could be attributed to the significance of the plastic surgery journals accepting their work. For example, the cohort of research fellows included in this study produced 85 clinical studies published in leading plastic surgery journals, including but not limited to *Plastic and Reconstructive Surgery* and *Aesthetic Surgery Journal* and 20 clinical studies published in prominent open-access journals such as *Aesthetic Surgery Journal Open Forum* and *Plastic and Reconstructive Surgery Global Open.* Additionally, 8 students contributed 30 publications to the *Aesthetic Surgery Journal* which has an Impact Factor of 3.30,^23^ reaching a larger audience and thus increasing the likelihood for citations. According to the aforementioned survey of ACAPS, program directors and faculty view plastic surgery research more favorably than publications under other topics and cite the “quality of journal publication” as most important when reviewing applications for residency.^1^ The steady increase in mean *h-*index could also be attributed to the quality of literature produced by fellows. Fellows with published works were 79.17% likely to have at least 1 citation; this number rises to 94.44% when fellows from 2021 to 2022 are excluded. This finding suggests their publications are well-written, align with current topics, and are quality contributions to the plastic surgery literature. Most importantly, the observed increase in mean *h-*index is likely attributable to the increase in publications and citations over time.

This analysis is most similar to that by Mehta, which used surveys administered to integrated plastic surgery applicants from 2013 to 2016 and ACAPS faculty to collect information about academic productivity during research fellowships and their perceived value.^1^ The authors’ study differs in that it is an attempt to measure academic output in the context of impact, thereby evaluating both the value of dedicated research fellowships and the importance of the work produced. These data were collected from bibliometric databases with peer-reviewed publications rather than survey data, which are subject to variations in interpretation and self-reporting, recall, and nonresponse biases.^18,19^ Carney quantified the academic research productivity of research fellows, residents, and faculty at the University of Pennsylvania Division of Plastic Surgery using *h*-index, number of peer-reviewed publications, and presentations.^24^ Their clinical fellowship supports residents as well as medical students, and they noted a significant increase in the overall productivity of the division since beginning their fellowship in 2008. Their results suggest that clinical fellows’ work benefits mentors, faculty, and the department/division at large, illustrating the extrinsic impact of fellows’ work. Although their thesis does not align precisely with the objectives of our study, the value of the research fellows’ productivity is notable.

Although all 26 research fellows included within this study ultimately matched into a United States residency position, 19 (73.08%) fellows matched to an integrated plastic surgery residency position. This is considerably higher than the 55.3% match rate for integrated plastic surgery reported in the 2022 NRMP match data.^25^ However, our finding of 73.08% is likely deflated given that a number of our research fellows chose to apply to other specialties and therefore did not participate in the match process for an integrated plastic surgery residency position. This highlights the immersive nature of the study institution's 3-pronged fellowship experience (research, didactic, and clinical). Likely, the nature of this fellowship provides students the opportunity to grasp the breadth and depth of plastic surgery and ultimately make more informed career decisions before the match process. Thus, structured research fellowships may play a role in decreasing integrated plastic surgery residency attrition rates by allowing students a glimpse into the day-to-day lives of integrated plastic surgery residents at high-volume institutions.

This bibliometric analysis is not without limitations. First, participation in conferences in the form of abstracts, posters, and presentations represents an important marker of scholarly output, especially for medical students. Indeed, the number of abstracts and presentations is factored into integrated plastic surgery applicants’ statistics; in 2020, the mean number of abstracts, presentations, and publications among integrated plastic surgery applicants was 19.2, the second highest across all specialties, surpassed only by applicants to neurological surgery.^26^ However, conference abstracts and presentations were excluded in this study because they are inconsistently published and therefore cannot be tracked using Web of Science (Clarivate), Open Research and Contributor ID (ORCID iD),^15^ ResearchGate,^16^ and Google Scholar^17^ which were the primary databases used to conduct this bibliometric analysis. The study was designed in this manner to avoid subjective self-reports from the fellows and therefore made capturing conference presentations/posters unfeasible. Second, our analysis would benefit from additional comparison with other institutions with dedicated research fellowships. It is also important to note that scholarly works produced by fellows with faculty not affiliated with the study institution were excluded in our analysis. Thus, although the total publications produced by this cohort are likely underestimated, this was done to simplify analysis and to highlight ongoing scholarly returns through work with a single academic institution. Future studies would help determine what types of research fellowships are most fruitful and have the most impact on a residency match in the desired field.

## CONCLUSIONS

This study describes a structured research fellowship for medical students in plastic surgery. It shows that the success of this clinical research fellowship is because of the careful integration of clinical experience, academic ownership, mentorship and modeling, and didactic engagement. The work conducted during the fellowship is not only immediately impactful but also has a cumulative influence as students transition to residency and ultimately practice and amass more citations and recognition for their contributions to the literature.
